# Contextualizing remote fall risk: Video data capture and implementing ethical AI

**DOI:** 10.1038/s41746-024-01050-7

**Published:** 2024-03-06

**Authors:** Jason Moore, Peter McMeekin, Thomas Parkes, Richard Walker, Rosie Morris, Samuel Stuart, Victoria Hetherington, Alan Godfrey

**Affiliations:** 1 Department of Computer and Information Sciences, Northumbria University, Newcastle upon Tyne, UK; 2 Nursing, Midwifery and Health, Northumbria University, Newcastle upon Tyne, UK; 3 Northumbria Healthcare NHS Foundation Trust, North Tyneside, Newcastle upon Tyne, UK; 4 Department of Sport, Exercise and Rehabilitation, Northumbria University, Newcastle upon Tyne, UK; 5 Department of Neurology, Oregon Health & Science University, Portland, OR USA; 6 Cumbria, Northumberland, Tyne and Wear NHS Foundation Trust, Wolfson Research Centre, Campus for Ageing and Vitality, Newcastle upon Tyne, UK

**Keywords:** Risk factors

## Abstract

Wearable inertial measurement units (IMUs) are being used to quantify gait characteristics that are associated with increased fall risk, but the current limitation is the lack of contextual information that would clarify IMU data. Use of wearable video-based cameras would provide a comprehensive understanding of an individual’s habitual fall risk, adding context to clarify abnormal IMU data. Generally, there is taboo when suggesting the use of wearable cameras to capture real-world video, clinical and patient apprehension due to ethical and privacy concerns. This perspective proposes that routine use of wearable cameras could be realized within digital medicine through AI-based computer vision models to obfuscate/blur/shade sensitive information while preserving helpful contextual information for a comprehensive patient assessment. Specifically, no person sees the raw video data to understand context, rather AI interprets the raw video data first to blur sensitive objects and uphold privacy. That may be more routinely achieved than one imagines as contemporary resources exist. Here, to showcase/display the potential an exemplar model is suggested via off-the-shelf methods to detect and blur sensitive objects (e.g., people) with an accuracy of 88%. Here, the benefit of the proposed approach includes a more comprehensive understanding of an individual’s free-living fall risk (from free-living IMU-based gait) without compromising privacy. More generally, the video and AI approach could be used beyond fall risk to better inform habitual experiences and challenges across a range of clinical cohorts. Medicine is becoming more receptive to wearables as a helpful toolbox, camera-based devices should be plausible instruments.

## Introduction

Contemporary research investigates free-living fall risk through habitual (community-based) monitoring with wearable inertial measurement units (IMUs), i.e., devices with accelerometers and/or gyroscopes across a wide range of different neurological conditions^1^. For example, examining habitual daily mobility through instrumented gait within Parkinson’s disease (PD) is notable for assessing abnormal step times (intrinsic digital-based bio-markers) to better understand underlying mechanistic limitations that may lead to a fall^1^. Free-living mobility assessment through instrumented gait (via IMUs) could improve targeted strategies for reducing falls, enabling personalized fall risk prevention^2^. For example, IMUs can measure abnormal spatial and temporal gait characteristics during ambulatory walks, including gait asymmetry or variability, which are associated with an increased risk of falls^3,4^.

The current limitation when using an IMU-based wearable alone is the lack of absolute contextual (extrinsic, environmental) information (e.g., where someone is walking), which could lead to inaccurate interpretations of gait abnormalities and incorrect fall risk assumptions. That limitation was described over a decade ago leading to a conceptual approach to better understand the powerful impact of environmental factors on gait and motor function in the home and community^5^. To overcome this, studies have begun to propose the use of e.g., smartphones or wearables with embedded global positioning system (GPS) functionality and/or embedded applications/apps to provide context such as weather and general environmental location^6^. However, those approaches cannot be used within buildings, and situations beyond indoor environments may rely on outdated maps. Moreover, those approaches fail to capture the granular/minute influences on gait, such as navigating raised pathways or gait variations due to ad-hoc/random obstacles (not easily determined from outdated maps), which could generate abnormal gait characteristics^7^. Furthermore, although the use of diary-based apps can enable self-report of contextual factors^8^, it is limited due to subjective reporting^9^. Therefore, recent research has explored the use of wearable cameras to augment IMU-based gait assessment to provide a more comprehensive understanding of fall risk beyond the lab^10^. However, there is taboo when using cameras and shunning of suggestions pertaining to their use in the home or community settings to capture real-world video data as ethical and privacy concerns are a primary and overriding rationale to avoid their deployment. For example, a wearable camera could capture sensitive information, such as details on a bank statement, personal letter, or images of children. Moreover, if cameras were to be used, they create pragmatic challenges for researchers to view and painstakingly categorize/label the video data. Specifically, video data needs to be annotated (labeled) with synchronized IMU data, dramatically increasing timelines for a complete fall risk assessment^5^. The ability to collect IMU gait with absolute contextual clarity on environmental factors would dramatically increase the understanding of fall risk at an individual level. However, the challenge is to routinely anonymize sensitive information contained within a video to uphold privacy while providing rich contextual information on the environment (for a comprehensive patient assessment).

Understandably, there is apprehension when suggesting video data capture, especially beyond the clinic and in the home or community. Ideally, any captured raw video data would not be seen first by any person (including any member of a person’s healthcare team) until necessary, e.g., data verification. Accordingly, perhaps the detection and obscuring of sensitive objects (e.g., people, letters) to uphold privacy prior to being seen is best facilitated with the routine use of artificial intelligence (AI)-based computer vision (CV). But what is that? Where AI enables computers to think, CV enables them to see, observe and understand, and derive meaningful information from images and videos^11^. Typically, CV is routinely discussed within the field of autonomous systems for applications in e.g., robot navigation^12^ or agriculture and food processing^13^. AI-based CV may provide very pragmatic insights to remotely assess fall risk while upholding privacy.

We posit that current CV approaches exist to uphold privacy and should be routinely harnessed. Granted, a multidisciplinary approach is required to ensure approaches are from best (computing) practices to ensure any development(s) are fit-for-purpose and robust to uphold privacy and ethical concerns. Here, this perspective adopts off-the-shelf approaches and showcases the use of an examplar deep learning model to anonymize sensitive information captured by a wearable camera to better inform IMU mobility-based gait characteristics. In doing so the suggested model will display how to preserve the contextual information for future consideration (in free-living/habitual fall risk assessment). Generally, the aim of this perspective is to suggest the routine use of accessible approaches that can be harnessed to enhance (enrich) personalized approaches in medicine.

### Proposed technologies

Here, we suggest a model that typifies how a CV approach would be created and adopted within the fall risk assessment. The aim here isn’t to champion the suggested model per se but to inspire the field of digital medicine to closely consider (reconsider, perhaps) the adoption of wearable cameras for free-living/habital data collection with the use of exemplar AI methods. The following sections detail a suggested approach by drawing upon the author’s experiences and then uses data collected within a single university setting only to showcase the application during a pilot test.

#### Building a reference

Typically, the first step in a custom CV model is the collection of an image-based dataset with (manual) annotation of (video-based) frames/images. That is a very time-consuming process especially when initiating a database, but the purpose is to train computers to recognize objects, classifying them from within an image. In contrast, openly available resources exist such as the Microsoft Common Objects in Context (COCO) dataset, containing approximately 330,000 images and >2.5 million object instances in 80 categories including those required for anonymization^14^. From the referenced work, Lin et al describe COCO not within the context of object recognition but rather scene understanding comparable to 91 object types that would be recognizable by a 4-year-old child.

#### Defining a model

The You Only Look Once (Yolo) series of algorithms are typically described as the cornerstones of object detection for scene understanding^15^. Through several iterations, the current state-of-the-art is Yolov8^16^, based on the Darknet-53 network architecture and using a similar approach to previous Yolo implementations but with improved detection and classification modules^17^. Specifically, the architecture of Yolov8 consists of a deep convolutional neural network (CNN) that can be trained on large datasets (such as COCO).

When an image is an input to Yolov8, it goes through a series of convolutional layers used to extract features from the image. The network then predicts a set of bounding boxes, each with a confidence score, that surround objects in the image. Yolov8 uses a single pass of the network to predict the bounding boxes and class probabilities directly from full images, eliminating the need for region proposal and feature alignment steps used in other object detection systems. The output of Yolov8 is a set of bounding boxes with confidence scores and class probabilities that represent the detected objects in the image. Those bounding boxes identify sensitive objects in the video.

#### Blurring sensitive objects

**Fig. 1:**
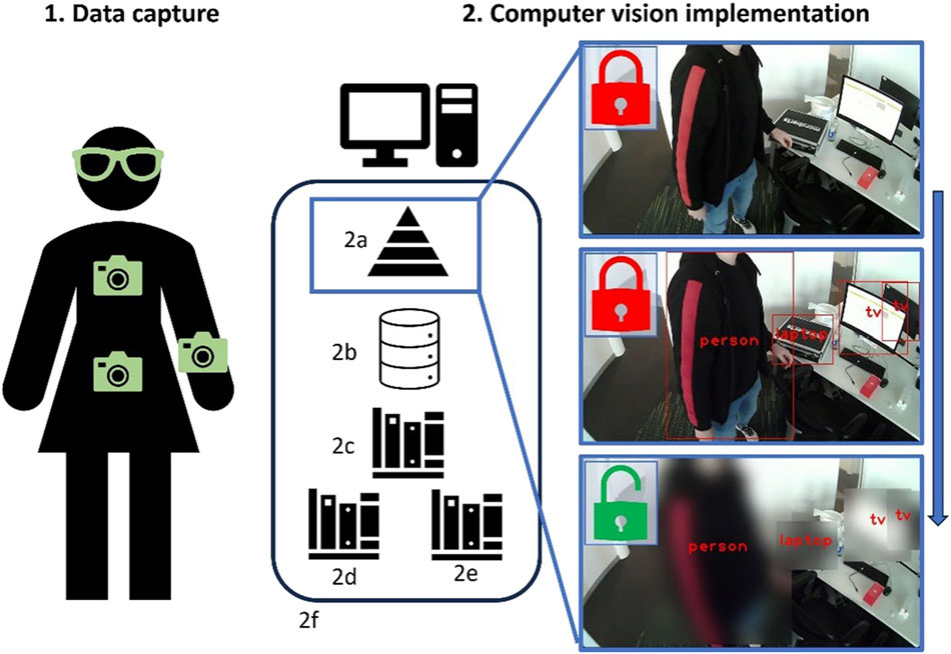
Video-based data capture could be gathered from any location via a wearable camera. Typically, common wear locations include the chest or waist (1). However, alternative locations with more routinely worn wearables could include the wrist (watch) or face/head via glasses (1). A CV model implementing YoloV8 (2a) drawing upon a well-characterized and comprehensive ground truth learning dataset/database (2b) and necessary libraries (2c, 2d, and 2e) via a suitable analytical environment (2f). The images to the right detail how the raw/original data (top) is anonymized with only the latter being visible as an output i.e., red locks indicate what is analyzed and then deleted with a green lock indicating the remaining image available for viewing. (A wearable IMU to quantify gait is worn on the lower back, not shown.) The algorithm selectively anonymizes only specific privacy-conscious objects such as screens, people, and documents while leaving the remaining content unanonymised to allow a better understanding of the environment in edge cases where the frame must be manually investigated.

A set of sensitive objects that should be obscured upon detection with habitual video capture were the pre-selected classes of *person*, *book*, *laptop*, and *TV*. Of those, many act as a catch-all for other objects (book: any text-based paper object, laptop: laptop or mobile phone, TV: any form of screen). When the Yolov8 model detects those objects in a video frame, a Gaussian blur filter is then applied using the *OpenCV* library for anonymization (Fig. 1). Specifically, the bounding box of the detected object is output by the Yolov8 model, and then the Gaussian blur is applied to that region of the video frame. To further ensure privacy an offset of 50 pixels was added to each side of the region of interest to improve privacy.

#### Video processing

Once all frames are processed, they are combined into a new anonymized video. A new video writer object is instantiated by *OpenCV* with each processed frame written before the output of the final anonymized video (*.avi*) before converting to *.mp4* due to its wider use and smaller file size.

#### Testing, a pilot study

To investigate the proposed model, 10 participants were recruited (M: 9, F: 1, 30.0 ± 6.3 years). Ethical consent was granted by the Northumbria University Research Ethics Committee (REF: 44692). Participants gave informed written consent before participating in this study. Testing took place within the City Campus, Northumbria University, Newcastle upon Tyne, UK.

To implement the model a Python 3.8 environment on a desktop containing an RTX 3070ti, Ryzen 7 3800X, and 24 GB RAM was chosen. *PyTorch* and *Ultralytics* libraries were used for manipulating tensors and accessing the YOLOv8 range of algorithms with the YOLOv8m model chosen for a good balance of speed and accuracy. The final model processes the video at 30 frames/second (fps).

#### Wearables: Video glasses and IMU

**Fig. 2:**
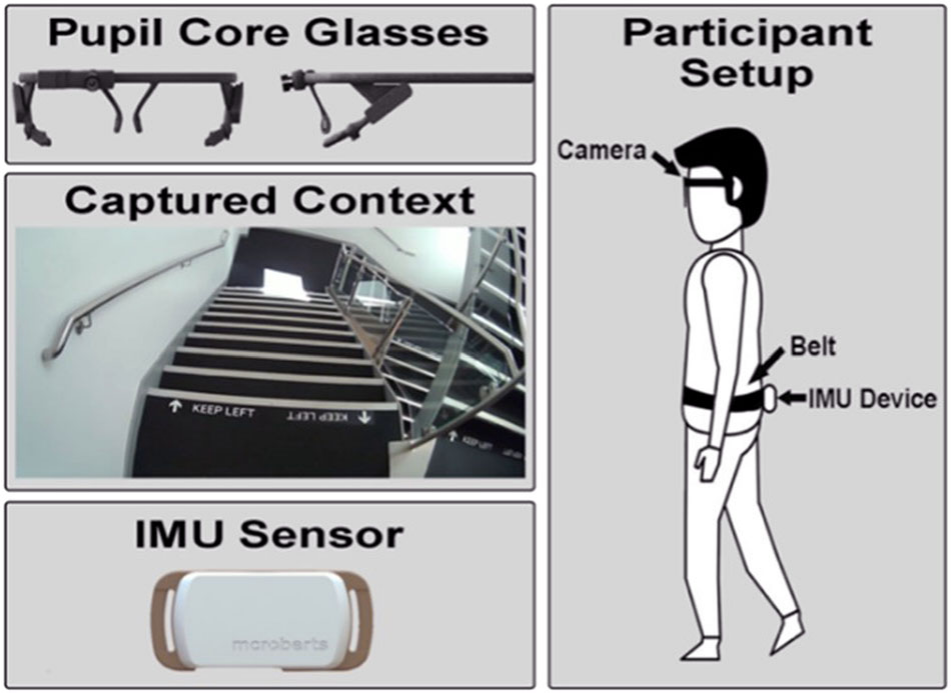
Video-based glasses showing visual perspective (captured context, in this example it is ascending indoor stairs) and IMU device along with a representation of both worn by a participant.

Any wearable camera and many attachment locations could be used within the context of gathering extrinsic data, but we suggest the use of wearable camera glasses as the technology is becoming more streamlined (i.e., subtle for daily use) and is typically ergonomically designed as well as being more user/patient friendly for passive sensing in comparison to a camera worn on the chest^18^. Wearable camera glasses also carry the potential of reduced injury in case of a fall event, especially when compared with a protruding chest-mounted camera that is likely to cause further injury to the participant upon impact. Moreover, camera-based glasses capture a participant’s visual perspective along with the wider/peripheral environmental context. Accordingly, each participant recruited wore the Pupil Labs Core wearable video glasses (https://pupil-labs.com/products/core/). The glasses feature three independent cameras consisting of a world (front-facing camera facing outwards) and two cameras facing inwards at each pupil to capture eye location. The world camera captures video at a resolution of 1920 × 1080 pixels at a frame rate of 30 Hz (Fig. 2) and is used here only. Audio data were automatically collected but removed upon video download.

Additionally, each participant wore a MoveMonitor IMU (McRoberts, 55 g, 106.6 × 58 × 11.5 mm) on a belt with the device on the lower back (specifically, at 5th vertebra level—L5) to collect inertial data (100 Hz). A validated algorithm was used to segment periods of walking from continuous inertial data^19^ stemming from the vertical acceleration used to identify the initial contact (IC) and final contact (FC) of each foot. For identification of IC and FC events a validated algorithm was used^20^ which filtered, integrated, and transformed the signal using continuous wavelet transforms (CWT). Once IC and FC times were found, temporal gait characteristics were computed. Spatial characteristics used another validated algorithm^21^, involving a high pass filter and double integration on the signal before using where *l* is IMU height from the ground and *h* is the absolute difference between the minima and maxima of the integration of the signal. The general methodology has been used for many years and is generally perceived as a pragmatic approach to habitual gait assessment^22,23^.

#### Protocol

Participants were asked to walk through naturally populated areas within the university campus. Participants navigated a range of environments including ascending and descending stairs, entering sensitive areas such as toilets, and having conversations with people. The video glasses and IMU were worn throughout the duration of the participant’s walk (approx. 10 min/participant), providing a continuous stream of synchronized data (via time stamps on a researcher’s computer) that captured the participant’s gait and environmental context.

#### Yolo evaluation metrics

When deciding on Yolo architecture (i.e., nano, small, medium, large, *x*-large) different evaluation metrics are available on the repository^16^ for the training results stemming from the COCO dataset^14^. Those metrics are:

- Size of the image being fed into the model, to ensure a fair test all model architecture fed the image in at a size of 640 × 640 px.
- Mean average precision (mAP50) value. Specifically, mAP50 is a measure of how accurate the predicted bounding boxes from the model are when compared with the manually labeled and drawn boxes of the objects within the dataset (ground truth data).
- Speed refers to the average inference time on a computer’s central processing unit (CPU) i.e., the time it takes to process an image through the network in milliseconds (ms).
- The number of parameters (params) present within the architecture, i.e., this is the total amount of different parameters that must be adjusted during training. This metric in essence gives insight into the size of the network.
- Floating point operations per second (FLOPs) which is a measure of the computational complexity of the model as it defines how many mathematical operations a model must do per second.

## Results

The YoloV8 model has previously been evaluated on the COCO dataset (Table 1, top), which contains 80 different object classes, including people, books, and electronics. Metrics suggest that the model is highly accurate and efficient, making it well-suited for the task of anonymizing videos. However, when assessing how well the model can truly generate privacy-sensitive videos mAP50 alone does not give a true indication of the effectiveness of the model for use in anonymization. This is due to the use of intersection over union (IoU) within mAP50. IoU is not a perfect measure of overlap, especially for objects with complex shapes like faces. IoU calculates the overlap between two bounding boxes as the area of the intersection divided by the area of the union. This means that even if a predicted bounding box is slightly off, it can still have a high IoU score if it covers most of the object. However, this does not mean that the object is truly anonymized, especially if the object is a face. While there may be a good overlap between predicted bounding boxes and the actual objects, if the box is even slightly off and allows for identification of a person’s face, it is not fit for purpose. Therefore, the only good metric is that of manual review, pertaining to how many sensitive data points are in the frame and how many of them are completely obscured.

**Table 1:** Architecture size metrics on COCO dataset and manual review anonymization review

**YOLOV8 Architecture size metrics**	**Model**	**Size**	**mAP50**	**Speed**	**Params**	**FLOPs**
	*YoloV8n*	640px	37.3	80.4	3.2	8.7
	*YoloV8s*	640px	44.9	128.4	11.2	28.6
	*YoloV8m*	**640px**	**50.2**	**234.7**	**25.9**	**78.9**
	*YoloV8l*	640px	52.9	375.2	43.7	165.2
	*YoloV8x*	640px	53.9	479.1	68.2	257.8
**YoloV8m Manual review anonymisation**	**Participant**	**People**	**Text**	**Screens**	**All**	
	*001*	0.988	1.00^a^	0.961	0.948	
	*002*	0.988	1.00^a^	0.972	0.959	
	*003*	0.989	1.00^a^	0.966	0.955	
	*004*	0.988	1.00^a^	0.952	0.940	
	*005*	0.983	0.914	0.944	0.848	
	*006*	0.986	0.926	0.938	0.856	
	*007*	0.982	0.933	0.943	0.863	
	*008*	0.986	0.928	0.939	0.859	
	*009*	0.988	0.874	0.929	0.802	
	*010*	0.984	0.934	0.933	0.857	
	Average	**0.986**	**0.918**	**0.947**	**0.887**	

^a^<20 frames of examples within the specific video.

To evaluate the effectiveness of the model, a sample of anonymized videos was manually reviewed, and it was found that the model was highly effective at obscuring sensitive data points such as faces, letters, and screens (Table 1, bottom). For the purposes of this perspective, to aid in categorizing the performance of the model the results were split into 3 categories (*people*: any person within the frame, *textual*: any form of text-based information like letters and bank statements *screens*: any form of the screen like televisions, laptops, or mobile phones). It was also found that the medium-sized model was capable of processing videos in real-time, making it a viable solution for real-world applications. In summary, the YoloV8 model was found to be a highly effective method of anonymizing videos, with the medium-sized model providing a good balance between performance and accuracy.

### Environmental context

**Fig. 3:**
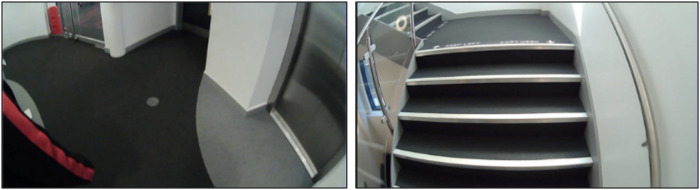
Flat-level terrain (left) and participant stair ascent (right).

To establish a baseline for natural gait, the study analyzed periods of the video in which no external factors affected the gait of the 10 participants (i.e., walks on flat/level ground). These baseline characteristics were then compared with the characteristics observed during periods when obvious external factors could influence gait, such as navigating stairs (Fig. 3). For instance when comparing baseline flat walking, stair ascent, and stair descent, the mean step, stance, stride, and swing times were all relatively similar. However, the standard deviation and asymmetry measures in these scenarios were found to differ significantly. For example, with one participant, when the context captured from the wearable video glasses showed flat-level terrain (Fig. 3) very low step time asymmetry (0.025) and standard deviation (0.019) values were observed (Table 2).

**Table 2:** Gait characteristics on different terrains

	Step time (s)	Stance time (s)	Stride time (s)	Swing time (s)	Step length (m)
*Flat-level*					
Mean	0.591	0.745	1.186	0.439	1.314
Asymmetry	0.025	0.030	0.023	0.029	0.051
Std. deviation	0.019	0.022	0.005	0.019	0.157
*Stair ascent*					
Mean	0.615	0.775	1.230	0.454	1.075
Asymmetry	0.054	0.052	0.011	0.029	0.210
Std. deviation	0.076	0.081	0.111	0.053	0.078

The resulting lower asymmetry (Asymmetry) and standard deviation (Std. deviation) from level flat terrain suggest a natural gait with no indication of fall risk. However, when the same participant’s gait is assessed a short time later during stair ascent (Fig. 3), there is a change in gait characteristics. For instance, we see step time asymmetry increase by 116% (0.025–0.054), and the standard deviation increases by 300% (0.019–0.076), Table 2.

These pilot results suggest that gait characteristics can be significantly affected by environmental factors and that clinicians must take these factors into account when evaluating fall risk. Without the use of video to supplement the numerical IMU gait characteristics with environmental context, clinicians may misinterpret data as an indication of increased fall risk caused by an intrinsic neurological disorder, such as PD, rather than a natural response to one’s environment.

## Discussion

The rationale for using wearables as objective tools to capture habitual longitudinal data to better understand personalized approaches in medicine is becoming profound^24^. There are many use cases and supporting arguments for adopting wearables to digitize traditional approaches in medicine to better inform clinical decision-making processes. Here, this perspective focuses on fall risk assessment through IMU-based wearables as they are a viable technology to passively capture robust and informative gait characteristics during everyday life across many cohorts^25^. Yet, they are limited by failing to provide absolute context, which could be exceptionally useful to enrich fall risk assessment (at an individual level). Accordingly, our perspective is that contemporary wearable cameras (especially glasses) and AI-based CVs should be now routinely proposed and used to *complete the picture* while simultaneously upholding privacy.

The next step for fall risk assessment beyond the lab/clinic is limited by two key challenges (i) privacy and (ii) technical integration. Perhaps both can be overcome by methods like the ones presented here. The proposed AI model in this pilot study is an example only to better inform fall risk assessment from combined wearables (video and IMU) data while preserving privacy. By automatically anonymizing video data, the AI provides a practical and efficient solution for protecting participant privacy while still enabling the collection of important contextual data. Understanding the environmental context, such as the presence of stairs or the type of flooring, is crucial to better understanding fall risk from habitual IMU gait assessment. Combining IMU data (i.e., validated gait characteristics) with the environmental context data (i.e., video-based data from ergonomically designed video capture devices like glasses), could better enable the understanding of factors (intrinsic and extrinsic) that contribute to fall risk in free-living.

Furthermore, the use of cameras in this study highlights the importance of considering the role of the physical (extrinsic) environment in fall risk assessment. While traditional laboratory-based assessments may be useful for evaluating specific aspects of gait and balance, they often do not replicate the complex and dynamic nature of real-world environments. The combination of wearable IMUs and video glasses, along with AI models (for anonymization), offers an appropriate and efficient approach for assessing fall risk in free-living environments.

Video analysis also needs to consider environmental factors that influence fall risk. For example, uneven terrain^26^, lighting, and obstacles can impact an individual’s mobility-based gait patterns that increase the likelihood of falls^23^. Previous studies have investigated that by using GPS sensors to infer terrain type, however, unlike video captured directly from the participant, absolute context cannot be gained from GPS sensors alone^27^. For example, GPS sensor data could be compared to freely available satellite images, but often they are outdated and so terrain types may be different. Equally, historical satellite images of outdoor environments cannot capture real-time or ad-hoc objects in the person’s daily path. Adoption of wearable video (to complement IMUs) would enable up-to-date terrain type classification with the detection of spontaneous obstacles.

Figure 1 displays the suggested scenario of how the AI proposed here could protect privacy (for the patient and bystander). The rationale for partial obfuscation is to ensure that any manual reviewing of a video frame upholds the clarity required to fully understand the environment. That approach is similar to previous work examining activity-orientated cameras to provide visual confirmation of specific activities from real-world settings^28^. By *keeping a human in the loop* it helps ensure visual confirmation to validate environmental circumstances. Of course, other camera sensing modalities could be used to further enhance approaches. An example includes a depth camera/sensor designed to determine the difference between the camera and the subject of an image which could offer further contextual information. Specifically, depth sensors are often used in combination with software algorithms to determine the outline of the subject (a person or other object) and apply a blur effect to the rest of the image. Such an approach in the context of free-living fall risk assessment may help determine a person’s adaptive gait to near and distant trip hazards. However, commercially available ergonomic glasses fitted with depth-sensing cameras are currently unavailable but related wearable concepts incorporating the technology have been proposed^29^.

Here, the suggested approach may be beneficial for clinical groups due to the potential usability of the glasses e.g., (i) prescription lenses can be included, and (ii) unobtrusiveness due to routine wearability compared to cameras mounted in other places. Furthermore, contemporary glasses with a smartphone app may reduce patient burden and minimize the risk of injury to patients e.g., if someone fell with a GoPro mounted on their chest, it would likely result in an injury to the sternum. The usability and safety considerations of wearable video glasses highlight their potential for practical application and wider clinical/medical implementations. In contrast, other generic camera-based technology (e.g., GoPro) may not be deemed fit for purpose due to their influence to increase social presence and social stigma, which could create social and surveillance discomfort for the wearer. Importantly, attempts to reduce any discomfort may result in behavior modification or abandoning the device^30^, negating the benefit of a remote, real-world patient assessment of fall risk.

Of course, cameras have been examined before in *life-logging* research to understand chronic disease self-management^31^. Previously, *life-logging* may have been perceived as a technical exercise rather than fulfilling an unmet clinic need, due to the use of readily identifiable cameras and manual processes needed over a prolonged period to gather, (patient) self-label and analyze data from daily events^32^. Thankfully, hardware has shrunk to be unobtrusive/discrete for continuous deployment (e.g., glasses) while software (e.g., Yolov8) and resources (e.g., COCO) are accessible and powerful to make laborious concepts^5^ more automated.

Although the patient-centric issue of technology interaction and privacy may be overcome with contemporary approaches, what about the bystander? Previously, *life-logging* human activity recognition (sitting, standing, walking) with an accelerometer and camera glasses was achieved while upholding bystander privacy via CNNs to uphold bystander annonymisation^33^. However, that work achieved a 70% accuracy only and was further limited with the adoption of a conservative a priori methodology, limiting the range of obfuscating in any environment. Interestingly, previous *life-logging* research has found that bystanders were generally accepting of the *life-logging* (i.e., camera) technology and life-logging (i.e., patients) engaged in propriety behaviors to help protect by stander privacy^34^. Accordingly, it could be assumed that for medical purposes use of camera-based technology to better inform fall risk could (i) be acceptable from bystanders e.g., friends and family in the home and (ii) empower the patient to remove cameras when s/he deems it necessary.

The pilot study to highlight the use of the suggested model was conducted with a small number of participants (*n* = 10) and all participants were tested within a modern university environment during daylight hours, which may limit the generalizability of the (AI-based) methodology to other settings and time of day. Regardless, the proposed approach and findings showcase the use of wearable cameras to inform IMU gait beyond lab settings, but there is much work to be done. Future research in this area must explore additional computer vision-based algorithms such as the inclusion of a sensitivity category (to complement the 3 suggested categories presented here). Specifically, locations pertaining to e.g., bathrooms, religious settings, and playgrounds are important to consider. One possible approach for a sensitivity category includes gathering original datasets from within the home and wider community to train existing models (e.g., YoloV8), obfuscating sensitive locations.

An unused feature of the wearable glasses presented here is eye-tracking. Harnessing the video data to inform IMU gait is a step forward for free-living fall risk assessment. Yet, the additional insight pertaining to participant gaze and specifically where s/he is looking during habitual walking would be a giant step forward for fall risk assessment. That is especially true in PD where the combined IMU and video-based eye-tracking glasses would provide a harmonious and holistic approach to provide a comprehensive understanding of vision and gait impairment pertaining to underlying mechanistic limitations^35^. For example, the referenced study draws attention to reduced saccade latencies and longer fixation durations during gait in PD which could be better explored in relation to fall risk during habitual data capture.

Although discussed within the context of fall risk with PD highlighted throughout, wearables (incorporating various sensing modalities including a camera) with AI-based CV contextualization (to automatically identify a range of objects, and items of interest to specific patient groups) could inform medical practice and routine care across a range of clinical cohorts. Hypothetical examples include (i) chronic obstructive pulmonary disease (COPD) and a better understanding of the environment influencing symptoms i.e., identification of air freshener plug-ins, weather, industrial vehicle exhaust^36^; (ii) obesity and automated food diary logging^37^ or; (iii) depression, where e.g., social context, activity, and location could influence symptoms^38^. Inter- and multidisciplinary fields need to ensure medicine is best equipped to comprehensively understand a person’s/patient’s living environment to inform better treatment.

## Conclusion

Apprehension often surrounds the use of video cameras in the home to capture patient data, fuelled by privacy and ethical concerns. However, wearable cameras could better inform free-living patient assessment, providing extrinsic (environmental) factors. Here, extrinsic video data from wearable glasses were used to better inform intrinsic digital bio-markers (i.e., IMU gait characteristics). Off-the-shield AI methods/resources could be harnessed to derive contemporary deep learning models to obscure/blur sensitive information and preserve contextual information necessary to better understand habitual patient data. In this perspective, AI was used to uphold privacy in video data to better understand abnormal gait indicative of elevated fall risk e.g., abnormal variability and asymmetry. Generally, video and AI have the potential to significantly improve the accuracy of habitual patient assessment while ensuring privacy and should be considered broadly for implementation across the field of digital medicine.

## Data Availability

Correspondence and requests for video and IMU data should be addressed to Alan Godfrey. Due to the nature of the data (raw video), access is limited and must be discussed on a per-project/access basis.

## Code availability

Please refer to https://github.com/JasonMooreNorthumbria for more information.
